# Psychological Resilience Among Older Adults with Physical Disability: A Scoping Review

**DOI:** 10.1093/geroni/igaf122.3365

**Published:** 2025-12-31

**Authors:** BoRin Kim, Sarah Sunghye Kang, Meagan Berry, Ke Li, Soobin Park, Sojung Park

**Affiliations:** University of New Hampshire, University of New Hampshire, New Hampshire, United States; University of California, Los Angeles, Los Angeles, California, United States; University of New Hampshire, Durham, New Hampshire, United States; Ohio University, Athens, Ohio, United States; Washington University in St. Louis, St. Louis, Missouri, United States; Washington University in St. Louis, St. Louis, Missouri, United States

## Abstract

With a growing number of older adults experiencing physical disability, understanding psychological resilience—an essential factor in adapting positively to these challenges—is crucial for promoting well-being in later life. However, limited research has specifically examined resilience among older adults with such disabilities. This scoping review aims to synthesize existing conceptual frameworks, measurement tools, and key contributing factors related to resilience in this population. Following PRISMA-ScR guidelines, a systematic search was conducted in PubMed, CINAHL, Web of Science, and PsycINFO for studies published from 2005 to 2024. Inclusion criteria encompassed older Americans aged ≥65 with physical disability not exclusively related to sensory or cognitive impairments, and an explicit focus on psychological resilience. Twenty-two articles were identified. Conceptual frameworks ranged from viewing resilience as an inherent personality trait to a dynamic process shaped by individual, interpersonal, and macro-level factors. Common measurement instruments included the Connor-Davidson Resilience Scale, Brief Resilience Scale, Ego-Resiliency Scale, and Hardy-Gill Resilience Scale. Factors consistently associated with resilience were optimism, positive affect, self-efficacy, social support, and adaptive coping strategies. Environmental facilitators, such as accessible housing and community resources, also emerged as significant. Overall, the findings underscore resilience as a multifaceted phenomenon, influenced by a dynamic interplay between personal attributes and broader sociocultural contexts. These insights highlight the need for multi-level interventions targeting psychological, social, and structural barriers. Future research should refine measurement tools and investigate how sociocultural contexts shape resilience, ultimately informing strategies to enhance well-being in this vulnerable population.

